# Novel pathogenic OCRL mutations and genotype–phenotype analysis of Chinese children affected by oculocerebrorenal syndrome: two cases and a literature review

**DOI:** 10.1186/s12920-021-01069-9

**Published:** 2021-09-06

**Authors:** Yu Zhang, Linxia Deng, Xiaohong Chen, Yingjie Hu, Yaxian Chen, Kang Chen, Jianhua Zhou

**Affiliations:** 1 Department of Pediatrics, Tongji Hospital, Tongji Medical College, Huazhong University of Science and Technology, No.1095, Jiefang Ave., Wuhan, 430030 People’s Republic of China; 2 Department of Endocrinology and Metabolism, Wuhan Children’s Hospital, Tongji Medical College, Huazhong University of Science and Technology, Wuhan, China

**Keywords:** Proximal tubulopathy, Lowe syndrome, *OCRL* gene, Splicing mutation, Deletion mutation

## Abstract

**Background:**

Oculocerebrorenal syndrome of Lowe is a rare X-linked disorder characterized by congenital cataracts, mental retardation, and proximal tubulopathy. This condition is caused by a mutation of *OCRL* gene (located at chromosome Xq26.1), which encodes an inositol polyphosphate 5-phosphatase.

**Case presentation:**

We identified two novel *OCRL* mutations in two unrelated Chinese boys, each with a severe phenotype of Lowe syndrome. A novel de novo deletion (hemizygous c.659_662delAGGG, p.E220Vfs*29) was present in patient 1 and a novel splicing mutation (hemizygous c.2257-2A > T) that was maternally inherited was present in patient 2. A renal biopsy in patient 2 indicated mild mesangial proliferative glomerulonephritis, mild focal mononuclear cells infiltration, and interstitial focal fibrosis. Moreover, renal expression of OCRL-1 protein in patient 2 was significantly reduced compared to a control patient with thin basement membrane disease.

**Conclusions:**

This study reports two novel *OCRL* variants associated with severe ocular and neurologic deficiency, despite only mild renal dysfunction. Based on our two patients and a literature review, the genotype–phenotype correlation of *OCRL* mutations with this severe phenotype of Lowe syndrome suggest a possible clustering of missense, deletion, and nonsense mutations in the 5-phosphatase domain and Rho-GAP domain in the Chinese population.

## Background

Oculocerebrorenal syndrome of Lowe (OCRL; OMIM #309000), also known as Lowe syndrome, is an X-linked recessive multisystem disorder in which patients present with major abnormalities of the eyes (congenital cataracts), the kidneys (renal tubular acidosis, Fanconi syndrome, renal rickets), and the central nervous system (hypotonia, areflexia, and mental retardation) [1, 2]. It is a rare disease with an estimated prevalence of about 1/500,000 in the general population. The manifestations of tubular dysfunction at onset vary among patients, and disease severity tends to increase with age. Proteinuria with low molecular weight (LMW) proteins is invariably present and aminoaciduria, hyperphosphaturia, bicarbonaturia, and hypercalciuria are frequently present. Progressive proximal tubulopathy ultimately leads to renal failure [2, 3].

This disease results from mutations of the *OCRL* gene (chromosome Xq26.1), which contains 24 exons and encodes a 105-kD phosphatidylinositol (4,5) biphosphate (PI(4,5)P2) 5-phosphatase [4, 5]. This protein is primarily located in the trans-Golgi network, endosomes, and endocytic clathrin-coated pits. A deficiency of this protein leads to dysregulation of vesicular transport, which may be decisive for the development of organ dysfunction that is typical of Lowe syndrome [6]. Most pathogenic mutations are deletions, frame-shifts, or premature termination codons; missense mutations and splice-site mutations are less frequent [7]. Here, we report two novel pathogenic DNA variations, a hemizygous deletion mutation c.659_662delAGGG and a hemizygous splicing mutation c.2257-2A > T, in two boys from China who had proximal tubulopathy and congenital cataracts due to Lowe syndrome, and discuss the genotype–phenotype correlations of this disease.

## Case presentation

### Case 1

A 5-year-old boy was referred to our department with proteinuria, delayed motor milestones, hypotonia, congenital cataract, and failure to thrive. His past medical history indicated he was born at full term after a normal pregnancy, received surgery for congenital cataracts at the age of 3 months, and experienced several limb fractures. He was the first child of a nonconsanguineous marriage, and detailed pedigree analysis indicated no family history of congenital disorders. At birth, his weight was 3200 g (-1SD-Median) and length was 51 cm (Median- + 1SD). The boy lifted his head at 8 months, sat up at 12 months, walked unassisted at 30 months, and talked at 3 years.

**Fig. 1:**
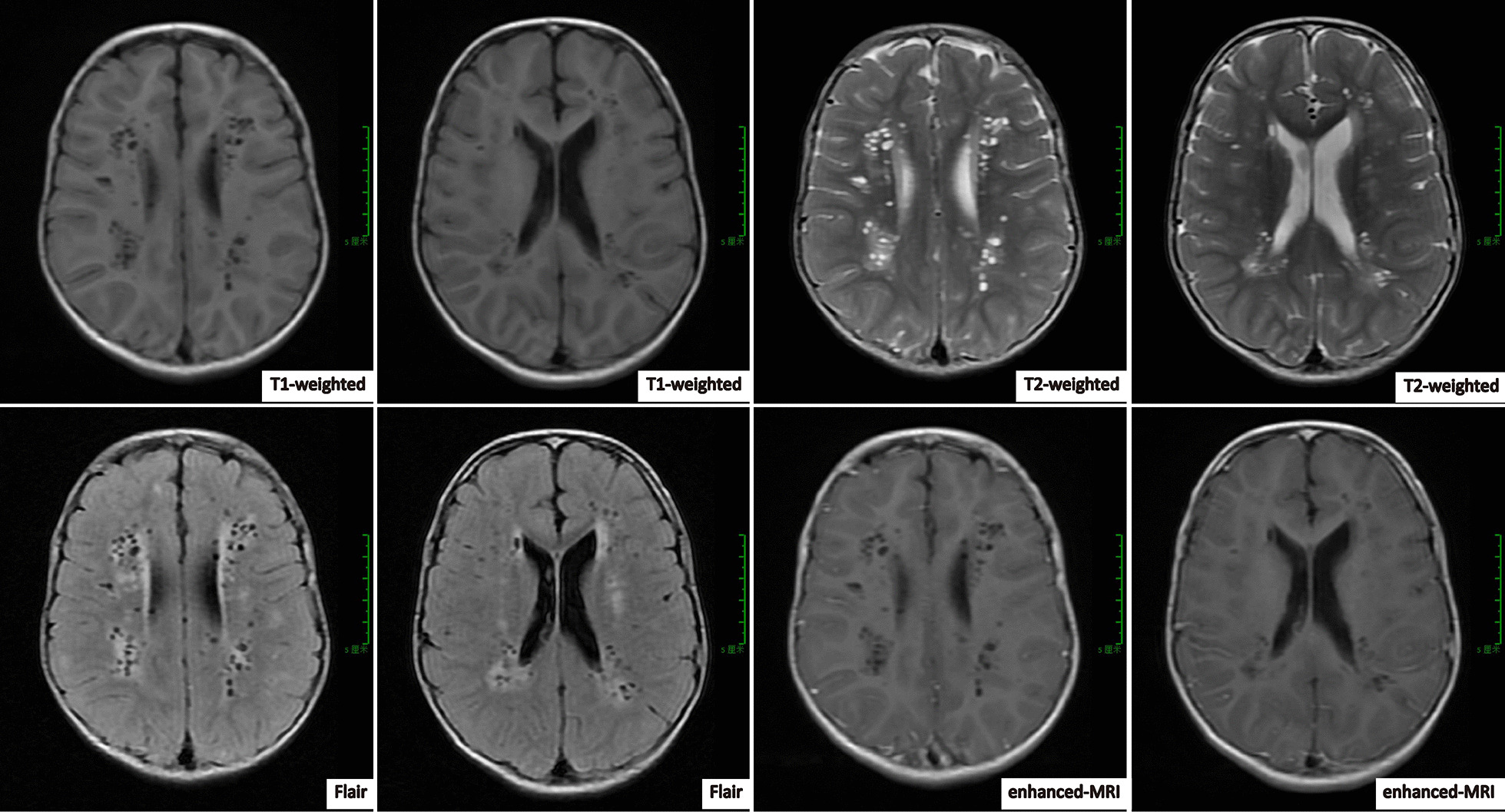
Brain MRI of patient 1 at the age of five years. Note the presence of ventriculomegaly, bilateral periventricular leukomalacia, and multiple gliotic lesions

The physical examination at presentation indicated his body weight was 13.3 kg (< -3SD) and length of 96 cm (< -3SD). He presented with slight frontal bossing, deep-set eyes, chubby cheeks, low-set ears, sparse hair, funnel chest, simian line, joint hypermobility, decreased muscle tone, and hyporeflexia. The laboratory findings indicated occasionally positive urine glucose, hypercalciuria, phosphaturia, mild proteinuria, β2-microglobulinuria, metabolic acidosis, and occasional elevations of creatinine kinase and lactate dehydrogenase (Table 1). The estimated glomerular filtration rate (eGFR), calculated using the Schwartz formula, was 162.6 mL/min/1.73 m^2^. Renal size and shape were normal based on an ultrasound examination. Magnetic resonance imaging (MRI) of the brain indicated mild ventriculomegaly, lacunar lesions in the bilateral white matter consistent with periventricular leukomalacia, and multiple gliotic lesions, which were hypointense on T1-weighted and hyperintense on T2-weighted analyses (Fig. 1). An electroencephalography examination indicated no abnormalities. He was attending a school for children with special needs because his IQ was 55 and he had serious sensory integrative dysfunction.

**Table 1:** Laboratory values of two patients with OCRL syndrome

Clinical parameter	Case 1	Case 2	Normal range
*Urine*			
pH	7.5	6.5	4.5–8.0
Glucose (quantitative)	Occasionally positive	Negative	Negative
Calcium (mmol/kg/24 h)	0.19	0.388	< 0.1
Phosphorus (mmol/kg/24 h)	0.87	0.52	0.5–0.6
Protein (mg/24 h)	624.7	226.4	≤ 140
β2-microglobulin (mg/L)	65.72	> 80	< 0.2
*Blood*			
HCO3- (mmol/L)	15.6	17.7	22.0–29.0
Calcium (mmol/L)	2.34	2.60	2.2–2.7
Phosphorus (mmol/L)	1.53	1.70	1.05–1.8
Creatinine kinase (U/L)	274	62	≤ 190
Lactate dehydrogenase (U/L)	455	382	120–300
25 (OH) vitamin D (ng/mL)	29.4	26.9	≥ 30
Urea (mmol/L)	4.28	2.00	1.7–8.3
Serum creatinine (μmol/L)	22	22	59–104
Uric acid (μmol/L)	183.4	162.0	202.3–416.5

**Fig. 2:**
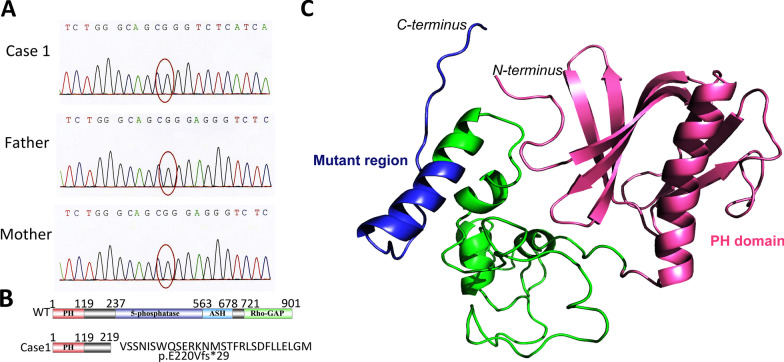
OCRL mutation in patient 1. **a** DNA sequencing of the *OCRL* gene indicated a novel 4-basepair deletion mutation (hemizygous c.659_662delAGGG, p.E220Vfs*29). **b** The 4-basepair deletion at exon 8 caused a frame shift after aa 220, resulting in a truncated protein of 247 aa. **c** Three-dimensional structural analysis of the mutated OCRL-1 protein

Whole-exome sequencing, with validation by Sanger sequencing, indicated a de novo hemizygous pathogenetic c.659_662delAGGG mutation in exon 8 of the *OCRL* gene (NM_000276.3; Fig. 2a), confirming the diagnosis. This deletion caused a frame shift from protein position 220 (between the PH and 5-phosphatase domains) leading to an insertion of 28 aberrant amino acids and a premature termination codon, resulting in a truncated protein of 247 out of 901 amino acids (p.E220Vfs*29; Fig. 2b) with an altered three-dimensional structure (Fig. 2c).

To date, he is receiving treatment with an angiotensin converting enzyme inhibitor (ACEI) for proteinuria, hydrochlorothiazide for hypercalciuria, a citrate mixture for metabolic acidosis, and rehabilitation training.

### Case 2

**Fig. 3:**
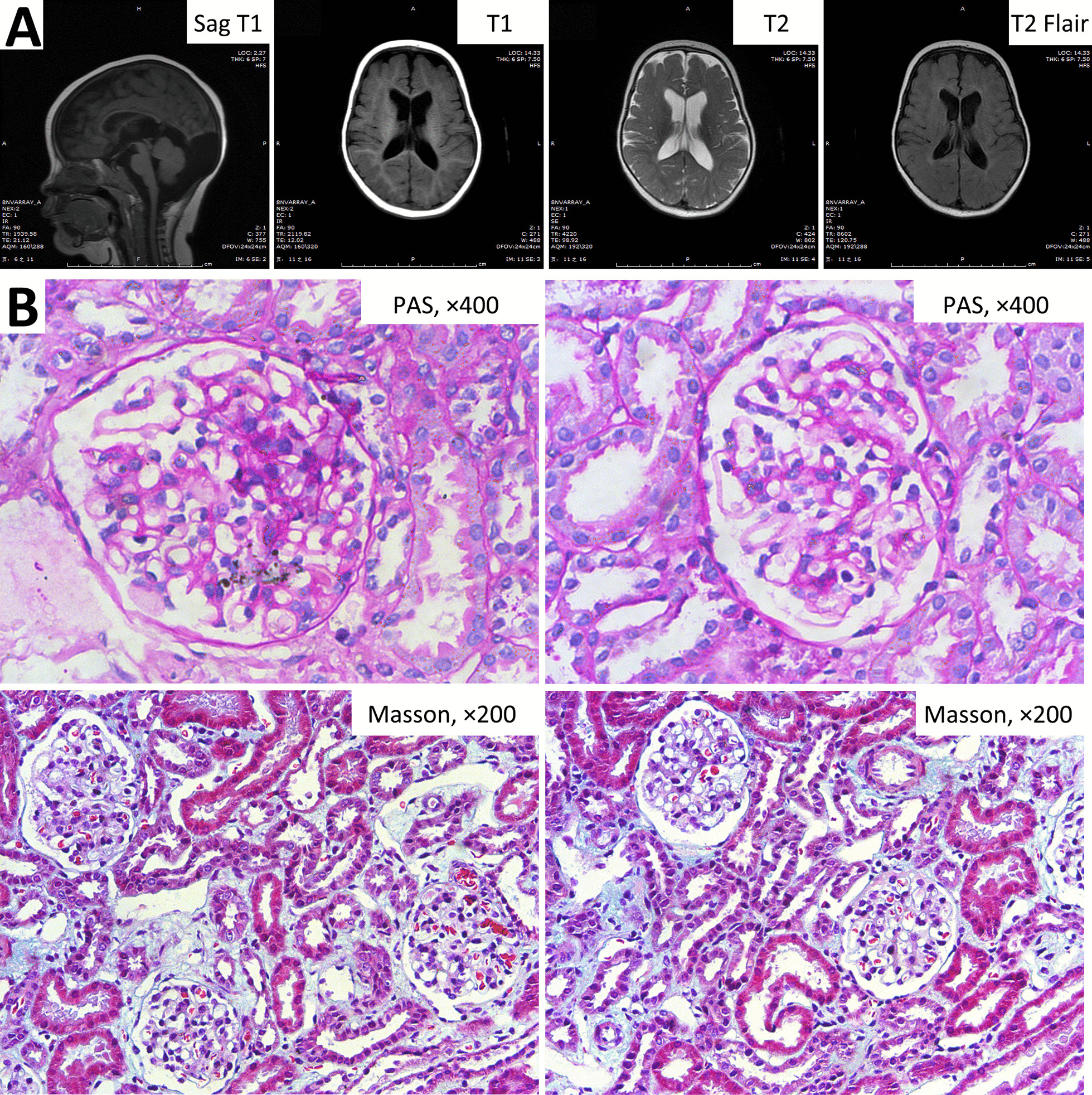
Brain MRI and renal pathological changes of patient 2 at the age of 7 months. **a** Note the presence of ventriculomegaly, white matter shrinkage, leukoaraiosis, cerebellar hypoplasia, and a large cistern magna cyst. **b** Note the presence of interstitial focal fibrosis with infiltration of focal mononuclear cells and mild mesangial proliferation

The boy, 7 months old, was the first baby of a healthy nonconsanguineous couple. The pregnancy was uneventful, the baby had spontaneous vaginal delivery, and his birth weight was 3450 g (Median- + 1SD). The boy had bilateral cataracts since birth, and underwent surgery at the age of 3 months, but his visual acuity remained poor. At the age of 7 months, he could not roll over or crawl, and could not sit without support. He was admitted to our hospital with proteinuria. At admission, physical examination indicated his weight was 11 kg (+ 2SD- + 3SD), height was 76 cm (+ 2SD- + 3SD), and that he had sparse hair, chubby cheeks, bilateral nystagmus, visual impairment, hypotonia, and absence of deep tendon reflexes. The laboratory findings indicated hypercalciuria, mild proteinuria, β2-microglobulinuria, metabolic acidosis, elevation of lactate dehydrogenase, and hypovitaminosis D (Table 1). The eGFR (Schwartz formula) was 137 mL/min/1.73 m^2^. An ultrasound examination indicated the size and shape of the kidneys were normal (left kidney: 6.5 × 2.5 cm, right kidney: 6.0 × 2.5 cm). MRI of the brain indicated severe ventriculomegaly, white matter shrinkage, leukoaraiosis, cerebellar hypoplasia, and a large cistern magna cyst (Fig. 3a). The electroencephalography results were normal. He could not complete the evaluation of psychomotor development because of poor vision. Histological examination of a renal biopsy sample indicated mild mesangial proliferation, mild infiltration of focal mononuclear cells, and interstitial focal fibrosis (Fig. 3b). Tubular lesions, including atrophy, necrosis, dilatation, and protein casts, were not detected.

**Fig. 4:**
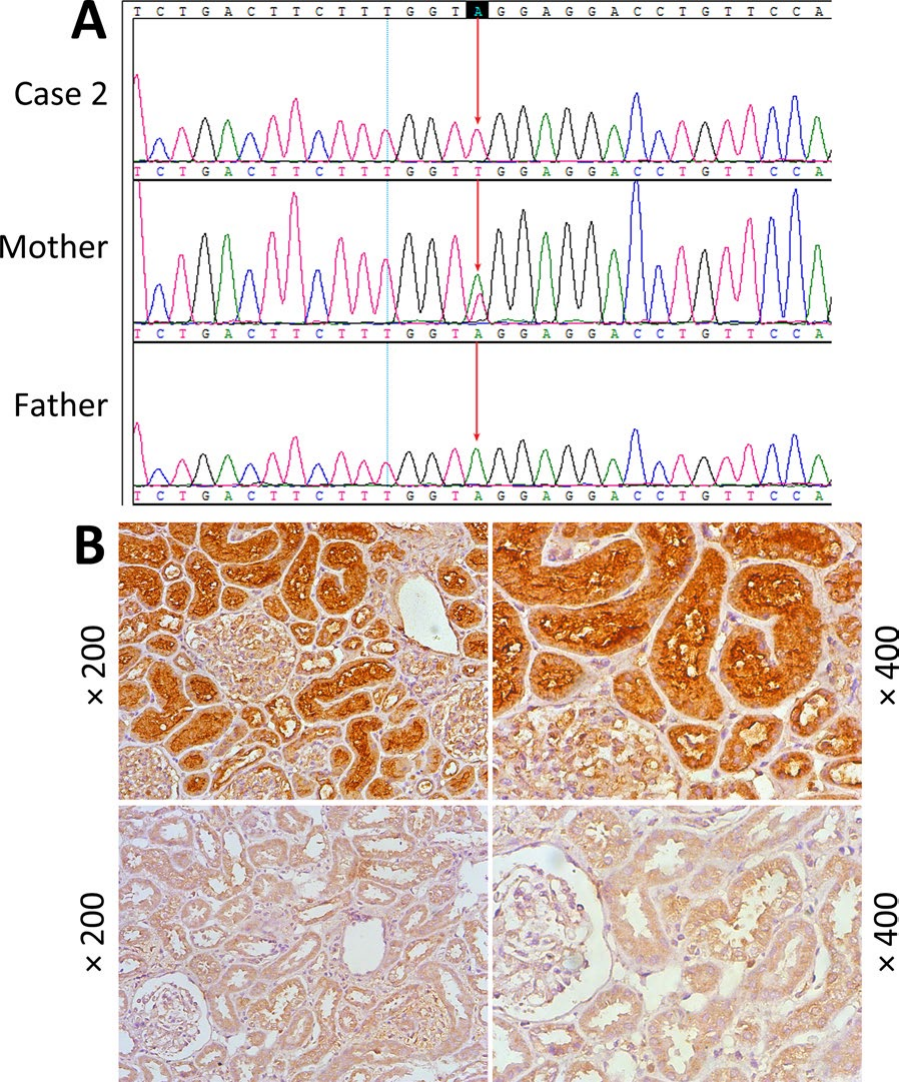
OCRL mutation in patient 2. **a** DNA sequencing of the *OCRL* gene indicated a novel splicing mutation (hemizygous c.2257-2A > T), which was inherited from his mother. The IVS20 sequencing result for the mother indicated a heterozygous mutation. **b** Renal immunohistochemical staining of OCRL-1 in patient 2 and a control patient with thin basement membrane disease. Patient 2 had a significantly reduced OCRL-1 protein level compared to the control

To confirm the clinical diagnosis of Lowe syndrome, we analyzed the *OCRL* gene. Whole-exome sequencing, with Sanger sequencing validation, indicated a hemizygous c.2257-2A > T DNA variant in *OCRL* intron 20 (NM_000276.3; Fig. 4a), a novel splicing variant. We then designed two pairs of primers for reverse transcription PCR of exon 8 and exon 12, but this yielded no transcriptional products. Therefore, we speculated that the mutated transcript was removed by nonsense mediated degradation (NMD) of the mRNA. We only detected the expression of OCRL-1 protein in renal tissues by immunohistochemistry using an anti-OCRL polyclonal antibody against the C-terminus of the human OCRL-1 protein (Proteintech Group, USA). This patient had a markedly reduced content of OCRL-1 in kidney tissue compared with a control patient with thin membrane basement disease (Fig. 4b). Thus, this splicing mutation in the *OCRL* gene led to almost no expression of the wild-type protein. The patient’s mother was heterozygous for the c.2257-2A > T mutation, but had no proximal tubulopathy, and refused an ophthalmologic examination.

We treated this patient with ACEI for proteinuria, a citrate mixture for metabolic acidosis, hydrochlorothiazide for hypercalciuria, intraocular lens implantation, and rehabilitation training to improve his motor and sensory integrative function.

## Discussion and conclusion

The clinical diagnosis of Lowe syndrome is based on specific ophthalmologic, renal and neurologic abnormalities [8]. Bilateral cataract is a hallmark finding among all clinical features. A history of cataract surgery during the early infantile period provided an important clue for the diagnosis of Lowe syndrome in the patients. The neurological features of Lowe syndrome are muscle hypotonia, areflexia, intellectual disability, seizures, behavioral abnormalities (e.g*.*, stubbornness, aggression, irritability, temper tantrums, complex repetitive purposeless movements, etc.), and abnormal findings in neuroimaging, such as brain atrophy, delayed myelination, mild ventriculomegaly, and hyperintense cerebral white matter on MRI T2-weighted images [9]. The two patients described here had the common clinical manifestations of muscle hypotonia, areflexia, and intellectual disability.

An MRI scan showed mild ventriculomegaly, bilateral periventricular leukomalacia, and multiple gliotic lesions in patient 1, and brain atrophy, leukoaraiosis, and ventriculomegaly in patient 2. Previous studies of affected patients reported the renal phenotype included LMW proteinuria, aminoaciduria, macroalbuminuria, hyperphosphaturia, hypercalciuria, glycosuria, metabolic acidosis, and progressive renal failure [7, 8]. Renal tubular dysfunction is not always present at birth, but usually manifests within the first few weeks to months of life. The onset of renal tubulopathy can be asymptomatic, but can also manifest as Fanconi syndrome or even chronic renal failure, and disease severity tends to increase with age. LWM proteinuria is a prominent finding and occurs in all patients, although aminoaciduria, bicarbonaturia, and hypercalciuria are also frequently present [6]. Both of our patients had incomplete Fanconi syndrome. Thus, tubular proteinuria, hypercalciuria, phosphaturia, metabolic acidosis, and focal interstitial fibrosis were present, but other renal tubular functions were normal. We therefore consider our patients to have a severe phenotype of Lowe syndrome.

Clinical findings are considered adequate for the diagnosis of Lowe syndrome, but gene analysis is necessary for genetic counseling and analysis of genotype–phenotype correlation. According to the Human Gene Mutation Database (http://www.hgmd.cf.ac.uk/ac/index.php), there are approximately 250 pathogenic DNA variations of *OCRL*, and more than 90% are in exon 10–18 and exon 19–23. Our patient 1 had a c.659_662delAGGG mutation in exon 8, which caused a frameshift and a premature termination codon at aa 248. Patient 2 had one splice-site mutation in intron 20, which presumably led to abnormal splicing of the RNA precursor and degradation of the mutated transcript by NMD. The two mutations identified here were not previously reported in any other patients with Lowe syndrome, and these two mutations seem related to a severe phenotype of Lowe syndrome.

To date, 30 *OCRL* mutations have been identified in Chinese patients with Lowe syndrome, including the two patients identified here (Table 2) [10–26]. Frameshift, splicing, or nonsense mutations leading to truncated proteins account for 67% of these mutations, missense mutations account for 27%, and genomic deletions account for 6%. Forty percent of these mutations occur in exons 9–16 (which encode the 5-phosphatase domain) and most of these mutations are single base pair missense changes. Another 40% of these mutations occur in exons 18–23 (which encode the RhoGAP-like domain in C terminus) and most of them cause truncated proteins. There was only one reported deletion mutation in exon 5 (which partially encodes the PH domain) and two reported mutations in exon 8 (which encodes the linkage domain between the PH and 5-phosphatase domains) that led to truncated proteins. Thus, based on literature review and our data, the genotype–phenotype correlation of *OCRL* mutations with the severe phenotype indicated clustering of frame-shifts and splicing mutations in or near the 5-phosphatase domain and the RhoGAP-like domain.

**Table 2:** OCRL mutations in Chinese patients with Lowe syndrome

Patient number	Mutation type	Exon/intron	Nucleotide change	Protein change	Literature
1	Missense	Exon 21	c.2290_2291delinsCT	p.Glu764Leu	Dai et al. [10]
2	Missense	Exon 23	c.2581G > A	p.Ala861Thr	Dai et al. [10]
3	Missense	Exon 14	c.1423C > T	p.Pro475Ser	Zheng et al. [11]
4	Missense	Exon 15	c.1502 T > G	p.Ile501Ser	Zheng et al. [11]
5	Nonsense	Exon 22	c.2464C > T	p.Arg822Ter	Zheng et al. [11]
6	Complex	Exon 22	c.2368_2368delG, c.2370A > C	p.Ala790Profs*34	Zhou et al. [12]
7	Microdeletion (249 kb)	–	[hg19]arrXq25q26.1 (128,652,372–128,901,629) × 0	–	Zhang et al. [13]
8	Deletion	Exon 14	c.1389delT	p.Phe463Leufs*57	Song et al. [14]
9	Nonsense	Exon 11	c.1000C > T	p.Arg334Ter	Gao et al. [15]
10	Nonsense	Exon 18	c.2083C > T	p.Arg695Ter	Gao et al. [15]
11	Nonsense	Exon 8	c.577G > T	P.Glu193Ter	Zhang et al. [16]
12	Microdeletion (633 kb)	–	[hg19]arrXq25q26.1 (128,155,802–128,789,721) × 0	–	Zhu et al. [17]
13	Nonsense	Exon 15	c.1528C > T	p.Gln510Ter	Gao et al. [18]
14	Insertion	Exon 20	c.2187dupG	p.Arg730Glufs*41	Gao et al. [18]
15	Nonsense	Exon 14	c.1366C > T	p.Gln456Ter	Gao et al. [18]
16	Missense	Exon 15	c.1499G > A	p.Arg500Gln	Gao et al. [18]
17	Deletion	Exon 13	c.1281_1282delTT	p.Cys428Hisfs*2	Li et al. [19]
18	Insertion	Exon 22	c.2367insA	p.Ala813Ter	Liu et al. [20]
19	Splicing	Intron 20	c.2437 + 2_2437 + 4delTAAinsC	–	Ji et al. [21]
20	Deletion	Exon 5	c.321delC	p.Leu108Serfs*29	Ji et al. [21]
21	Complex	Exon 8 Exon 22	c.562C > T c.2464C > T	p.Leu188Phe p.Arg822Ter	Chen et al. [22]
22	Missense	Exon 15	c. 1571A > G	p.His524Arg	Shi et al. [23]
23	Deletion	Exon 18	c.1897delT	p.Ser633Leufs*11	Zhang et al. [24]
24	Deletion	Exon 15	c.1470delG	p.Lys491Asnfs*29	Zhang et al. [24]
25	Missense	Exon 15	c.1538A > G	p.Tyr513Cys	Zhang et al. [24]
26	Nonsense	Exon 10	c.880G > T	p.Glu294Ter	Ke et al. [25]
27	Insertion	Exon 24	c.2626dupA	p.Met876Asnfs*8	Ke et al. [25]
28	Insertion	Exon 20	c.2146_2147insTT	p.Ser716Phefs*27	Chen et al. [26]
29	Deletion	Exon 8	c.659_662delAGGG	p.E220Vfs*29	This report
30	Splicing	Intron 20	c.2257-2A > T	–	This report

In summary, we identified two patients with novel *OCRL* variants who had severe ocular and neurologic deficiency, although only mild renal dysfunction. This study also emphasizes that a genotype–phenotype correlation of *OCRL* mutations with a severe phenotype of Lowe syndrome due to clustering of missense, deletion, and nonsense mutations in the 5-phosphatase domain and the Rho-GAP domain in the Chinese population.

## Data Availability

The raw sequence data reported in this paper have been deposited in the Genome Sequence Archive (Genomics, Proteomics & Bioinformatics 2017) in National Genomics Data Center (Nucleic Acids Res 2021), China National Center for Bioinformation/ Beijing Institute of Genomics, Chinese Academy of Sciences, under accession number HRA001169 that are publicly accessible at https://ngdc.cncb.ac.cn/gsa-human/browse/HRA001169.

## Abbreviations

OCRL: Oculocerebrorenal syndrome of Lowe
PI(4,5)P2: Phosphatidylinositol 4,5-bisphosphate
HGMD: Human gene mutation database

## References

[1] Lowe CU, Terrey M, MacLachlan EA (1952). Organic-aciduria, decreased renal ammonia production, hydrophthalmos, and mental retardation; a clinical entity. AMA Am J Dis Child.

[2] Loi M (2006). Lowe syndrome. Orphanet J Rare Dis.

[3] Zaniew M, Bokenkamp A, Kolbuc M, La SC, Baronio F, Niemirska A, Szczepanska M, Bürger J, La MA, Miklaszewska M (2018). Long-term renal outcome in children with OCRL mutations: retrospective analysis of a large international cohort. Nephrol Dial Transpl.

[4] Zhang X, Jefferson AB, Auethavekiat V, Majerus PW (1995). The protein deficient in Lowe syndrome is a phosphatidylinositol-4,5-bisphosphate 5-phosphatase. Proc Natl Acad Sci USA.

[5] Suchy SF, Olivos-Glander IM, Nussbaum RL (1995). Lowe syndrome, a deficiency of a phosphatidylinositol 4,5-bisphosphate 5-phosphatase in the Golgi apparatus. Hum Mol Genet.

[6] Mehta ZB, Pietka G, Lowe M (2014). The cellular and physiological functions of the lowe syndrome protein OCRL1. Traffic.

[7] Hichri H, Rendu J, Monnier N, Coutton C, Dorseuil O, Poussou R, Baujat G, Blanchard A, Nobili F, Ranchin B (2011). From Lowe syndrome to dent disease: correlations between mutations of the OCRL1 gene and clinical and biochemical phenotypes. Hum Mutat.

[8] Bokenkamp A, Ludwig M (2016). The oculocerebrorenal syndrome of Lowe: an update. Pediatr Nephrol.

[9] Allmendinger AM, Desai NS, Burke AT, Viswanadhan N, Prabhu S (2014). Neuroimaging and renal ultrasound manifestations of Oculocerebrorenal syndrome of Lowe. J Radiol Case Rep.

[10] Dai C, Wang L, Li Y, Zheng Z, Qian J, Wang C, Liu Z, Shan X (2019). Lowe syndrome with extremely short stature: growth hormone deficiency may be the pathogeny. Growth Factors.

[11] Zheng B, Chen Q, Wang C, Zhou W, Chen Y, Ding G, Jia Z, Zhang A, Huang S (2019). Whole-genome sequencing revealed an interstitial deletion encompassing OCRL and SMARCA1 gene in a patient with Lowe syndrome. Mol Genet Genomic Med.

[12] Zhou FQ, Wang QW, Liu ZZ, Zhang XL, Wang DN, Dongye MM, Lin HT, Chen WR (2019). Novel mutation in OCRL leading to a severe form of Lowe syndrome. Int J Ophthalmol-Chi.

[13] Zhang Y, Li R, Jing X, Tang X, Li F, Liao C (2019). Clinical and molecular genetic analysis of a pediatric patient with Lowe syndrome. Zhonghua Yi Xue Yi Chuan Xue Za Zhi.

[14] Song Y, Li G (2019). A case of infant lowe syndrome. Shangdong Da Xue Xue Bao (Yi Xue Ban).

[15] Gao S, Li J, Lin J (2019). Two cases of children lowe syndrome. Sichuang Da Xue Xue Bao (Yi Xue Ban).

[16] Zhang C, Wang S, Chen R, Zheng L, Liao Z (2017). A case of oculo-cerebro-renal syndrome in adult. Zhongguo Shen Jing Mian YI Xue He Shen Jing Bing Xue Za Zhi.

[17] Zhu X, Li J, Ru T, Zhu RF, Dai CY, Wang WJ, Hu YL (2017). Prenatal diagnosis and follow-up of a case with Lowe syndrome caused by interstitial deletion of Xq25-26. Zhonghua Yi Xue Yi Chuan Xue Za Zhi.

[18] Gao Y, Jiang F, Ou ZY (2016). Novel OCRL1 gene mutations in six Chinese families with Lowe syndrome. World J Pediatr.

[19] Li B, Zhang Z, Zhou Q, Yang J, Wu X, Liu G (2016). Clinical features and OCRL mutation analysis in a case of infant Lowe syndrome. Chin J Pathophysiol.

[20] Liu T, Yue Z, Wang H, Tong H, Sun L (2015). Novel mutation of OCRL1 in Lowe syndrome. Indian J Pediatr.

[21] Ji L, Chen C, Li H, Du P (2015). Lowe syndrome with novel OCRL mutations in Chinese children: report of two cases. J Clin Pediatrics.

[22] Chen Q, Chen Y, Zhang W (2015). A case of Lowe syndrome. Zhonghua Er Ke Za Zhi.

[23] Shi RM, Bian XH, Li LM, Liu XH (2014). Investigation and OCRL mutation analysis of a family with oculocerebrorenal syndrome of Lowe. Zhongguo Dang Dai Er Ke Za Zhi.

[24] Zhang YQ, Wang F, Ding J, Yan H, Yang YL (2013). Novel OCRL mutations in Chinese children with Lowe syndrome. World J Pediatr.

[25] Ke YH, He JW, Fu WZ, Zhang ZL (2012). Identification of two novel mutations in the OCRL1 gene in two Chinese families with Lowe syndrome. Nephrology (Carlton).

[26] Chen S, Zhang X, Chen L, Tian Q, Jiang W (2014). Analysis of OCRL gene mutation in a male infant with Lowe syndrome. Zhonghua Yi Xue Yi Chuan Xue Za Zhi.

